# Editorial: Vitamin D: from pathophysiology to clinical impact

**DOI:** 10.3389/fnut.2025.1572567

**Published:** 2025-03-13

**Authors:** Cristina Vassalle

**Affiliations:** Fondazione G. Monasterio, Pisa, Italy

**Keywords:** hypovitaminosis D, vitamin D, 25(OH)D, extraskeletal diseases, vitamin D status

Beyond its well-established role as a key factor in calcium and phosphate homeostasis and bone health maintenance, vitamin D has been recognized as a key determinant for many extraskeletal targets (1). Indeed, while the calcium ion itself is involved in a large number of cellular and metabolic pathways, vitamin D interacts (negatively or positively) with hundreds of genes, which greatly expands its spectrum of action (2). In particular, the numerous non-classical effects of vitamin D include the regulation of cellular proliferation, differentiation, apoptosis, and immunity (3). Accordingly, a variety of conditions, including but not limited to infections, diabetes, cardiovascular and neurodegenerative diseases and cancer, all of which are of public health concern, have been found to be related to reduced 25(OH)D levels (4). This fact is also important in light of the fact that hypovitaminosis D, which is generally considered to be present when 25(OH)D values—the most reliable serum biomarker of vitamin D status—are < 75 nmol/L (30 ng/ml), is prevalent worldwide, especially among the poor, dark-skinned individuals, children and the elderly along with obese subjects. Levels of 25(OH)D are affected by the environment (especially seasonal variation, geographical latitude, and sun exposure), to a lesser extent by diet (food and supplementation), and also by one's own genetic background (1).

The manuscripts included in this Research Topic contribute to the ongoing debate on the determinants of vitamin D status, the effects of inappropriate vitamin D levels on many extraskeletal conditions and overall and specific mortality, along with supplementation issues (Table 1). Because diet is only a minor source of vitamin D (few foods provide significant amounts of vitamin D), supplementation is an easy and low-cost strategy to restore adequate 25(OH)D levels and reduce the risk of adverse health outcomes, and one contribution to this Research Topic evidenced comparable efficacy of intermittent vs. cumulative vitamin D supplementation in improving 25(OH)D concentrations (Table 1). Moreover, the genetic causal association between some diseases and the circulating levels of vitamin D was investigated in some contributions through Mendelian randomization, a methodology that uses genetic variation as an instrumental variable to assess the association with specific outcomes of interest (Table 1) (5).

**Table 1:** Contributions to the Research Topic “*Vitamin D: From Pathophysiology to Clinical Impact*.”

**Title**	**Authors**	**Setting**	**Patients**	**Results**
The roles of serum vitamin D and tobacco smoke exposure in insomnia: a cross-sectional study of adults in the United States	Gao T, Hou M, Wang Q, Liu D, Chen F, Xing Y, Mei J	Association between 25(OH)D, smoking and insomnia	Adults	25(OH)D (< 75 nmol/L) may affect the association between tobacco smoke exposure and insomnia; however, vitamin D may also affect insomnia in non-smokers
Determinants of cancer incidence and mortality among people with vitamin D deficiency: an epidemiology study using a real-world population database	Lai YC, Chen YH, Liang FW, Wu YC, Wang JJ, Lim SW, Ho CH	Population of vitamin D deficient subjects	Adults	Vitamin D deficiency in patients with liver disease was associated with an increased incidence of cancer; subjects with dementia had an increased mortality rate among cancer patients. Additionally, older age and diabetes mellitus were associated with both increased cancer incidence and mortality
Could vitamin D concentration be a marker of a long hospital stay in older adults patients?	Nowak J, Jabczyk M, Jagielski P, Hudzik B, Brukało K, Borszcz J, Zubelewicz-Szkodzińska B	Hospitalization length and vitamin D status	Elderly subjects (>60 years)	Patients with 25(OH)D < 31.2 nmol/L had a 47% higher risk of prolonged hospitalization (>11 days)
Efficacy of intermittent versus daily vitamin D supplementation on improving circulating 25(OH)D concentration: a Bayesian network meta-analysis of randomized controlled trials	Zhuang Y, Zhu Z, Chi P, Zhou H, Peng Z, Cheng H, Xin X, Luo W, Si S, Mo M, Chen D, Liu H, Yu Y	Vitamin D supplementation;116 randomized controlled trials (*n* = 11,376 participants)	Adults	The efficacy of intermittent vitamin D supplementation in improving circulating 25(OH)D levels at equivalent cumulative dosage and duration is similar to that of daily supplementation
Circulating vitamin levels mediate the causal relationship between gut microbiota and cholecystitis: a two-step bidirectional Mendelian randomization study	Miao C, Xiao L, Xu X, Huang S, Liu J, Chen K	The role of vitamin D in the relationship between the gut microbiota and cholecystitis (Mendelian randomization study design)	Adults	Vitamin D may mediate the relationship between gut microbiota and cholecystitis
Vitamin D, vitamin D supplementation and atrial fibrillation risk in the general population: updated systematic review and meta-analysis of prospective studies	Ding X, Lai J, Zhang H, Guo Z	Atrial fibrillation risk; meta-analysis (seven studies)	Adults	Vitamin D deficiency/insufficiency is associated with an increased risk of atrial fibrillation in the general population
Maternal vitamin D status and risk of childhood overweight at 5 years of age in two Nordic cohort studies	Amberntsson A, Bärebring L, Winkvist A, Lissner L, Meltzer HM, Brantsæter AL, Papadopoulou E, Augustin H	Pediatric overweight subjects	Children	Maternal 25(OH)D < 30 nmol/L, particularly in mothers with overweight or obesity, predicted lower BMI in their 5-year-old children, but not the risk of being overweight
Causal effect of vitamin D on myasthenia gravis: a two-sample Mendelian randomization study.	Fan Y, Huang H, Chen X, Chen Y, Zeng X, Lin F, Chen X	Subjects with myasthenia gravis (Mendelian randomization study design)	Adults	No clear causal relationship was found between vitamin D deficiency and myasthenia gravis
Three-stage pattern of rapid increase, plateau, and subsequent decline in vitamin D concentration during pregnancy among Chinese women: a large-scale survey	Wang H, Zhang F, Li B, Fu M, Shan X, Ma Y	Pregnant subjects	Adult pregnant women	Trend in 25(OH)D during pregnancy (Three-stage pattern: increase, plateau, and decline)
Vitamin D status, sleep patterns, genetic susceptibility, and the risk of incident adult-onset asthma: a large prospective cohort study	Chang Q, Zhu Y, Zhou G, Liang H, Li D, Cheng J, Pan P, Zhang Y	Individuals with asthma risk	Adults	Increased serum vitamin D levels were associated with a lower risk of incident adult-onset asthma, and this association was modified by sleep patterns and genetic predisposition to asthma
The effects of vitamin D on all-cause mortality in different diseases: an evidence-map and umbrella review of 116 randomized controlled trials	Cao M, He C, Gong M, Wu S, He J.	Risk of mortality and vitamin D status; analysis of 116 randomized controlled trials (*n* = 149, 865 participants)	Adults	Vitamin D supplementation may reduce respiratory cancer mortality in patients with respiratory cancer and all-cause mortality in COVID-19 and patients with liver disease
A meta-analysis of the association between vitamin D supplementation and the risk of acute respiratory tract infection in the healthy pediatric group	Fang Q, Wu Y, Lu J, Zheng H	Subjects with acute respiratory tract infection	Children	No clear benefit was found for vitamin D supplementation in reducing acute respiratory tract infections
Vitamin D status and tic disorder: a systematic review and meta-analysis of observational studies	Xiaoxia L, Jilong J, Xianrui C, Yanhui C	Tic disorders and vitamin D status (metanalysis of 13 observational studies)	Children and adolescents	Vitamin D levels in children with tic disorder were lower than in controls
Prevalence of vitamin D deficiency and associated risk of all-cause and cause-specific mortality among middle-aged and older adults in the United States	Wang TY, Wang HW, Jiang MY	Risk of overall and specific mortality and vitamin D status	adults	Vitamin D deficiency, but not insufficiency, correlated with increased mortality risk
Causal associations between insulin-like growth factor 1 and vitamin D levels: a two-sample bidirectional Mendelian randomization study	Gou Z, Li F, Qiao F, Maimaititusvn G, Liu F	Associations between insulin-like growth factor 1 and vitamin D in the general population (two-sample bidirectional Mendelian randomization study)	Adults	IGF-1 has positive causal and reverse causal relationships with Vitamin D and serum 25-OHD, respectively
Associations between vitamin D levels and dietary patterns in patients with Hashimoto's thyroiditis	Kaličanin D, Cvek M, Barić A, Škrabić V, Punda A, Boraska Perica V	Hashimoto's thyroiditis		Cofee and sugar intake can adversely affect vitamin D status, whereas other food groups have positive associations (e.g. vegetables)
Serum 25-hydroxyvitamin D as a predictive biomarker of clinical outcomes in patients with primary membranous nephropathy	Duan S, Chen S, Lu F, Zhou M, Jiang L, Chen C, Geng L, Sun R, Xu Y, Huang Z, Zhang C, Zhang B, Mao H, Xing C, Yuan Y	Primary membranous nephropathy (PMN)	Adults	25(OH)D was significantly correlated with nephrotic proteinuria and anti-PLA2R Ab seropositivity in PMN and was also a predictor of remission outcomes
Results of longitudinal Nutri-D study: factors influencing winter and summer vitamin D status in a Caucasian population	Hribar M, Pravst I, Pogačnik T, Žmitek K	Determinants of vitamin D status in the general population	Adults	In addition to seasonal variations, personal factors such as BMI, skin pigmentation, vitamin D intake, and protective behavior against the sun are important determinants of vitamin D status
Correlation between hyperlipidemia and serum vitamin D levels in an adult Chinese cohort	Wang J, Shi T, Xu L, Li Y, Mi W, Wang C, Lu P, Li L, Liu Z, Hu Z	Relationship between dyslipidemia and vitamin D status in the general population	Adults	The prevalence of hyperlipidemia is higher in the population with low 25(OH)D, and lower 25(OH)D significantly increases the risk of total cholesterol and triglyceride alterations

It is true that at present it has not been definitively proven that vitamin D has an effective causal role in many human pathophysiological conditions or that low vitamin D is only an epiphenomenon, given the controversial aspects that remain to be resolved (e.g. limited sample sizes, differences in ethnicity, variations in vitamin D dosage, adopted reference limits for defining inadequate levels of vitamin D, and unmeasured confounding factors) and the underlying mechanisms by which vitamin D may affect so many biological processes that remain largely to be better understood. Nonetheless, given the widespread involvement of vitamin D in key biological processes, it seems biased to think of vitamin D only as a marker of disease. However, the question remains: how can the suboptimal global distribution of a single vitamin cause the risk of so many diseases? The triage theory, proposed by Ames in 2006, offers a mechanistic explanation for the relationship between chronic and even moderate deficiency of a micronutrient, such as vitamin D, and multiple chronic degenerative conditions related to aging, such as neurodegenerative and cardiometabolic diseases and cancer, in terms of a “triage” response to micronutrient undersupply (6). Indeed, in the case of a reduced intake of micronutrients, this theory proposes that micronutrients may be shifted for short-term survival to maintain pathways critical for life, at the expense of other, less essential processes, but which insidiously increase the risk of developing chronic degenerative conditions in the long term. And why can one person's risk for a particular disease be higher than another's? Vitamin D is not the only micronutrient that matters; more than 30 vitamins and essential minerals/elements have been identified as critical factors in reducing the risk of onset and development of chronic degenerative diseases and in extending life expectancy and quality. In addition to vitamin D, other essential vitamins include A, B (e.g. B1, B2, B3, B6, B9, B12), C, E, K, and minerals/elements such as calcium, chloride, iodine, iron, manganese, magnesium, phosphorus, potassium, selenium, sodium, and zinc; other key biomarkers may still be unknown or neglected (2). It is possible that the reduced combination of micronutrients generates a profile that may mark the fate of the development of a certain pathophysiological condition over another one. Accordingly, maintaining/restoring adequate levels of these factors could be a pillar of “personalized preventive medicine” and allow enormous savings in healthcare spending. In recent years, the complexity of the effects derived from the modulation of these dietary biomarkers and the discovery of possible further key determinants can be confronted with the development of large-scale technologies that allow the simultaneous acquisition of transcriptome, epigenome, proteome, and metabolome data that can be merged (e.g. omics data integration, network analysis, and machine learning). However, the challenge always remains great, requiring multiple skills and expertise in metabolism, nutrition, biochemistry and regulatory functions and collaboration between different professionals in basic and clinical areas. Moreover, translating research data into clinical applications and policy will not be simple; the achievement of this target will represent one of the biggest challenges of biological and medical research in the next few years. Nonetheless, the many recent advances and efforts in this field will allow us to identify critical pathways and biomarkers and to develop individualized patterns for dietary responses in a new approach that will be helpful to expand future beneficial and sustainable nutritional strategies to safeguard health and reduce the risk of disease, modulated to the real individual needs of each subject.

The wide variety of article types, methodologies, and designs of the studies included in this Research Topic evidences the interest and the potential impact of this topic on future healthcare and personalized preventive medicine, and it underlies the need for further efforts to expand our knowledge and overcome all the gaps and challenges that still exist in this research area.
